# A case of giant cell tumor of the breast, clinically suspected as malignant breast tumor

**DOI:** 10.1186/s40792-019-0635-4

**Published:** 2019-05-10

**Authors:** Mitsuo Terada, Naomi Gondo, Masataka Sawaki, Masaya Hattori, Akiyo Yoshimura, Haruru Kotani, Yayoi Adachi, Madoka Iwase, Ayumi Kataoka, Kayoko Sugino, Makiko Mori, Nanae Horisawa, Yuri Ozaki, Hiroji Iwata

**Affiliations:** Department of Breast Oncology, Aichi Cancer Center, 1-1, Kanokoden, Chikusa-ku, Nagoya city, Aichi 464-8681 Japan

**Keywords:** Giant cell tumor, Giant cell tumor of soft tissue, Breast tumor, Metaplastic carcinoma

## Abstract

**Background:**

Giant cell tumor (GCT) of the breast is scarce. We report a case of GCT of the breast which was suspected as a malignant breast tumor.

**Case presentation:**

A 74-year-old woman noticed a tender lump in her right breast. We suspected a malignant tumor spreading widely with axillary lymph node metastasis on clinical examination and imaging. Histological evaluation of the biopsy tissue revealed a tumor composed the proliferation of oval to spindle-shaped cells and multinucleated giant cells without malignant epithelial cells. The tumor cells stained positively for CD68 and negatively for estrogen receptor, progesterone receptor, and human epidermal growth factor receptor 2. The pathological findings suggested GCT, and fine needle aspiration biopsy for the axillary lymph node was negative. However, there was a gap between the clinical presentation, such as a tender mass suggesting rapid growth and multiple lymphadenopathies, and the pathological presentation of biopsy, which made us hesitate to conclude GCT as the final preoperative diagnosis. We could not rule out the possibility of malignant tumors with OGCs before surgery. We performed mastectomy and sentinel lymph node biopsy according to a surgical procedure for node-negative breast cancer with a wide ductal spread. The resected tissue histologically showed the same findings to the biopsy tissue. The definitive diagnosis of GCT of the breast was given, because the tumor lacked epithelial components, marked cellular atypia, and pleomorphism.

**Conclusions:**

GCT of the breast occasionally pretends as breast malignant tumors. Complete tumor resection should be performed for local control and the definitive diagnosis.

## Background

Giant cell tumor of soft tissue (GCT-ST) is uncommon and defined as a primary soft tissue neoplasm that is histologically and clinically similar to GCT of the bone [1]. In 1972, Salm and Sissons [2] firstly reported GCT-ST as a case series of ten cases of soft tissue tumors that had histologic features identical to those of GCTs of the bone. Above all, GCT-ST of the breast is rare, and only a few cases were reported in the medical literature [3–6]. The histological feature is composed of a mixture of round and oval mononuclear cells and multinucleated osteoclast-like giant cells (OGCs) with a blood vessel-rich stroma. Most of GCT-ST and GCT of the bone follow a benign clinical course, sometimes locally aggressive, and rarely metastasize. We report a case of GCT of the breast, which was clinically suspected as a malignant tumor.

## Case presentation

**Fig. 1:**
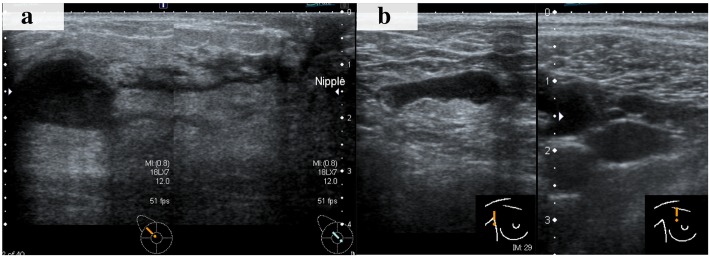
Ultrasound (US) showed an 18 × 16-mm, irregular-shaped, and hypoechoic mass with a suspicion of a spread to the nipple inside the duct (**a**). One of the swollen lymph nodes in her right axilla (**b**)

**Fig. 2:**
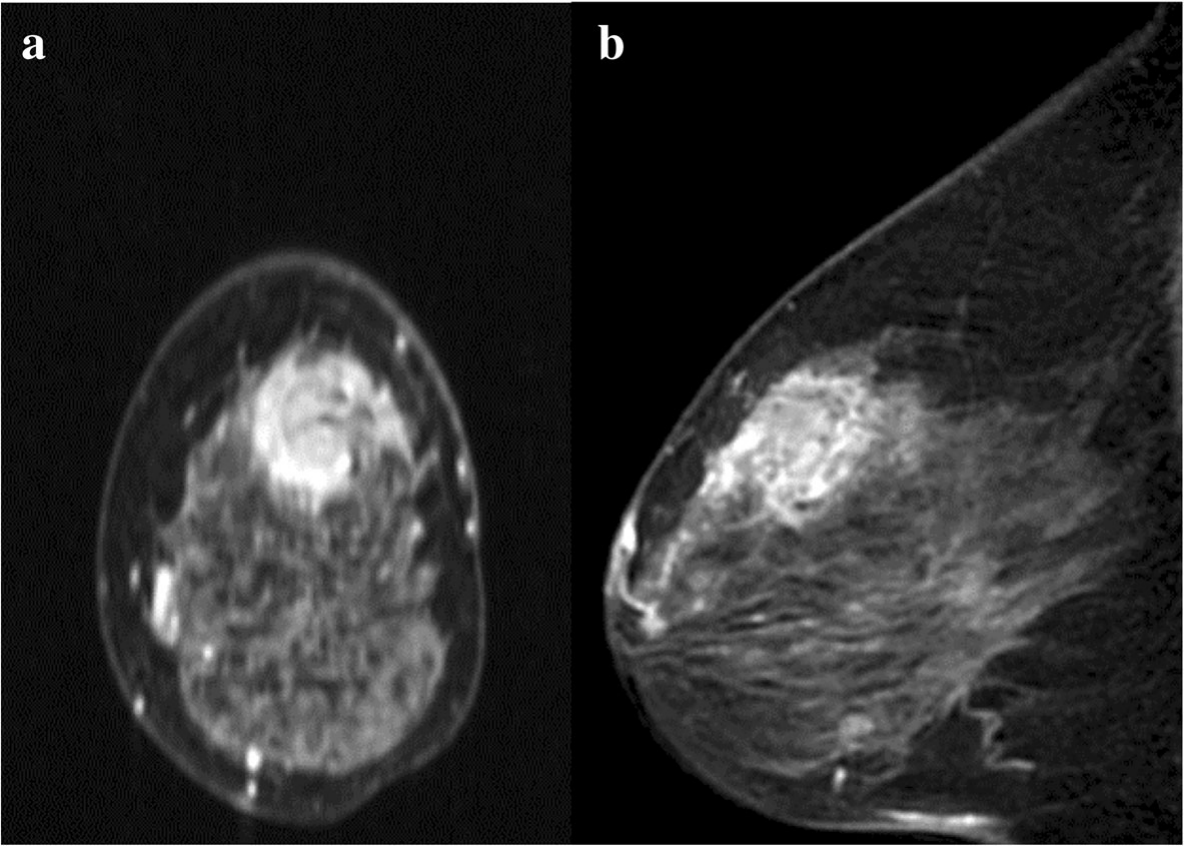
Magnetic resonance imaging (MRI) showed enhancement of a 17 × 17-mm indistinct mass surrounded with a non-mass enhanced segmental lesion toward the nipple side spreading a maximum of 74-mm range. Coronal view (**a**). Sagittal view (**b**)

**Fig. 3:**
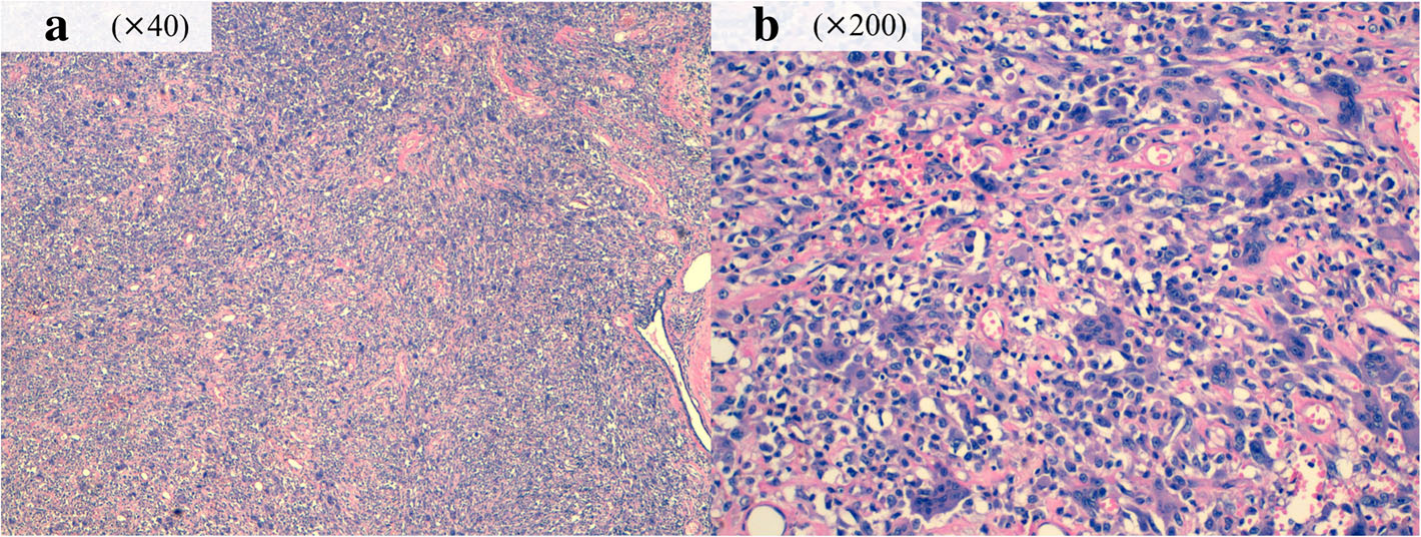
Histology of the surgical specimen. The tumor was composed mainly of oval and spindle mononuclear histiocyte-like cells and multinucleated giant cells without malignant cells; hematoxylin and eosin stain, × 40 (**a**) and × 200 (**b**)

**Fig. 4:**
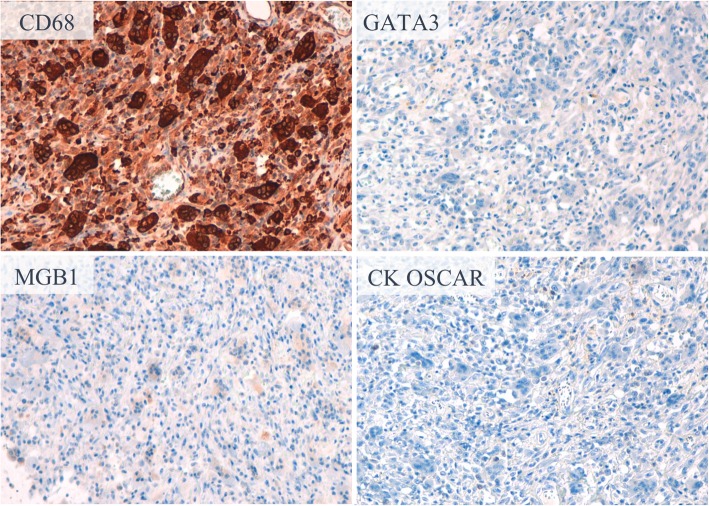
Immunohistochemistry of the tumor. Oval and spindle mononuclear histiocyte-like cells and multinucleated giant cells stained positively for CD68 and negatively for GATA3, MGB1, and CK OSCAR

A 74-year-old Japanese woman noticed a tender lump in her right breast. She immediately went to a breast clinic to get a breast cancer screening. She had no family history of breast and ovarian cancer. After a month, she was referred to our institution with suspicion of metaplastic breast carcinoma with a core needle biopsy at the breast clinic. Physical examination revealed a hard, tender, and 25-mm mass in the upper outer quadrant of her right breast and a palpable lymph node in her right axilla. Mammography indicated an indistinct mass on the mediolateral oblique view and the craniocaudal view. Ultrasound (US) showed an 18 × 16-mm, irregular-shaped, and hypoechoic mass with a suspicion of a spread to the nipple inside the duct (Fig. 1a) and several swollen lymph nodes in levels I to II (Fig. 1b). Magnetic resonance imaging (MRI) detected enhancement of a 17 × 17-mm indistinct mass surrounded with a non-mass enhanced segmental lesion toward the nipple side spreading a maximum of 74-mm range, which had no interaction with the chest bone, muscles, and breast skin, in the right breast tissue (Fig. 2). Invasive carcinoma with multiple axillary lymph node metastases was strongly suspected on clinical examination and imaging. Histological evaluation of the biopsy for the mass revealed a tumor with the growth of oval and spindle-shaped cells and multinucleated giant cells, the infiltrating lymphocyte into the breast tissue, and hyalinization in the stroma. The multinucleated giant cells stained positively for CD68. A part of the oval and spindle mononuclear cells stained weakly positive for CD68. These tumor cells stained negatively for estrogen receptor (ER), progesterone receptor (PgR), and human epidermal growth factor receptor 2 (HER2). There was a focal hemorrhage without necrosis. Few non-epithelial atypical cells were observed in the breast duct, but no atypical epithelial cells consistent with breast cancer were detected. GCT of the breast, breast cancer with OGCs, and giant cell-rich sarcomas should have to be considered as differential diagnoses, and the pathological findings suggested most GCT. Fine needle aspiration biopsy for the swollen lymph node revealed only normal lymphocyte, even though metastatic lymph node was strongly suspected on US. However, we could not rule out the possibility that the biopsy tissue showed a part of malignant tumor with OGCs and biopsy for the lymph node was false negative, because there was a gap between the clinical presentation, such as a tender mass suggesting rapid growth and multiple lymphadenopathies, and the pathological presentation of biopsy tissue. To obtain further evidence of malignancy, the tumor was sampled using a vacuum-assisted US-guided biopsy again. The result was the same as the prior biopsy. After discussing the treatment plan with the patient, we performed mastectomy and sentinel lymph node biopsy according to a surgical procedure for node-negative breast cancer with a wide ductal spread. The resection tissue histologically revealed similar findings to the biopsy specimen. The tumor was composed mainly of oval and spindle mononuclear histiocyte-like cells and multinucleated giant cells (Fig. 3). The mitotic figure of these cells did not stand out. There was no evidence of malignancy, and only intraductal epithelial hyperplasia around the tumor, which did not fill the criteria of ductal carcinoma in situ (DCIS). No sentinel lymph nodes contained malignant cells, and we concluded the lymphadenopathies were a response to the inflammation around the tumor. Immunohistochemically, a high proportion of the multinucleated giant cells stained positively for CD68 (Fig. 4). A part of the oval and spindle mononuclear cells stained weakly positive for CD68. These cells were negative for CK OSCAR, GATA-3, and MGB1 (Fig. 4). These findings were consistent with the GCT of the breast. The patient received no adjuvant therapy because GCT-ST is usually considered as a benign tumor. She is being followed up with regular clinical examinations without any symptoms of recurrence after 1 year past from surgery.

## Discussion

We reported a rare GCT of the breast case, which needed to be differentiated with malignant tumors. GCT of the breast was firstly reported in 1981 [6]. Histologically, GCT of the breast is composed of proliferating round to spindle mononuclear cells and multinucleated OGCs similar to GCT of the bone. The OGCs exhibit strongly positive staining for CD68, histocyte-specific marker, and the mononuclear cells are focally positive, while negative for epithelial markers [7]. To the best of our knowledge, there have been only four case reports of GCT of the breast [3–6], and the natural history and clinical course have not been well revealed yet. In one case, the tumor rapidly enlarged to 13 cm with palpation in a few months [6]. Another case finally underwent mastectomy for local recurrence after excisional biopsy despite having a GCT of the breast diagnosis with the excisional biopsy [4]. On the other hand, only one case had a definitive diagnosis with needle biopsy and omitted resection with no growth after biopsy [5]. All of the cases including ours were suspected malignancy with image findings.

Although GCT-ST is usually considered as a benign tumor, the prognosis is uncertain, and the standard therapy of GCT of the breast has not been established. As stated above, GCT of the breast can be potentially recurrent, and it suggests that inadequate resection can cause a local recurrence. Therefore, it is our firm belief that complete resection should be performed for GCT of the breast. In our case, we considered mastectomy as a reasonable procedure to resect completely, because MRI suggested that the tumor spread widely. Partial mastectomy can be acceptable, only when the tumor is localized enough to achieve complete resection. Sentinel lymph node biopsy can also be acceptable, only when breast cancer is suspected on clinical examination and imaging.

The rareness and the malignant-mimicking clinical presentation may cause difficulty in diagnosis. The differential diagnosis includes breast cancer with OGCs, giant cell-rich leiomyosarcoma, giant cell-rich osteosarcoma, malignant fibrous histiocytoma, and metastatic GCT of the bone [4]. Most of the breast cancers with OGCs are moderately or poorly differentiated invasive ductal carcinoma [8]. Metaplastic carcinoma with OGCs, a rare subtype and reported 11% of metaplastic carcinoma [9], may require to be differentiated with GCT of the breast [10], because histological characteristics of metaplastic carcinoma with OGCs are a dominant stromal component-containing OGCs and the bland-appearing spindle cell stroma presents various features, occasionally positive for CD68 [8]. GCT of the breast can be differentiated with them because it lacked epithelial component, marked cellular atypia, and pleomorphism [3]. In our case, the pathological assessment for the surgical specimen revealed the relatively homogeneous and bland-appearing feature and the lack of marked nuclear pleomorphism through the whole tumor, and these findings ruled out breast cancer with OGCs, leiomyosarcoma, osteosarcoma, and malignant fibrous histiocytoma. The absence of a history of GCT of the bone ruled out metastatic GCT of the bone.

## Conclusion

We presented a rare tumor case of GCT of the breast. Complete tumor resection should be performed for local control and the definitive diagnosis. Awareness of GCT of the breast is essential, and careful long-term follow-up is needed to understand the clinical course of GCT of the breast.

## Abbreviations

DCIS: Ductal carcinoma in situ
ER: Estrogen receptor
GCT: Giant cell tumor
GCT-ST: Giant cell tumor of soft tissue
HER2: Human epidermal growth factor receptor 2
MRI: Magnetic resonance imaging
OGCs: Osteoclast-like giant cells
PgR: Progesterone receptor
US: Ultrasound
